# Assessment of a three-axis on-rotor sensing performance for machining process monitoring: a case study

**DOI:** 10.1038/s41598-022-21415-w

**Published:** 2022-10-07

**Authors:** Chun Li, Zhexiang Zou, Kaibo Lu, Hongjun Wang, Robert Cattley, Andrew D. Ball

**Affiliations:** 1 School of Industrial Automation, Beijing Institute of Technology, Zhuhai, 519088 People’s Republic of China; 2 Centre for Efficiency and Performance Engineering, University of Huddersfield, Huddersfield, HD1 3DH UK; 3 College of Mechanical Engineering, Taiyuan University of Technology, Shanxi, 030024 People’s Republic of China; 4 School of Mechanical and Electrical Engineering, Beijing Information Science and Technology University, Beijing, 100192 People’s Republic of China

**Keywords:** Engineering, Electrical and electronic engineering, Mechanical engineering

## Abstract

Online monitoring of cutting conditions is essential in intelligent manufacturing, and vibrations are one of the most effective signals in monitoring machining conditions. Generally, traditional wired accelerometers should be installed on a motionless or stable platform, such as a tool holder or lathe bed, to sense vibrations. Such installation methods would cause the signals to suffer more serious noise interferences and a low signal-to-noise ratio, resulting in less sensitivity to valuable information. Therefore, this study developed a novel three-axis wireless on-rotor sensing (ORS) system for monitoring the turning process. The Micro Electromechanical System (MEMS) accelerometer sensor node can be mounted on a rotating workpiece or spindle rotor and is more sensitive in detecting the vibrations of the entire rotor system without any modification of the lathe system and interference in the cutting procedure. The processor, data acquisition, and Bluetooth Low Energy (BLE) 5.0+ modules were developed and debugged to cooperate with a piezoelectric triaxial accelerometer, with a vibration amplitude not larger than ± 16 g. A series of turning tests were conducted and the results were compared with those from the commercial wired accelerometers, which proved that the ORS system can measure the vibration signal of the rotor system more effectively and sensitively than wired accelerometers, thus demonstrating the accurate monitoring of machining parameters.

## Introduction

Cutting is one of the most essential and fundamental manufacturing technologies^1^. Online monitoring of the cutting process is essential for improving production efficiency, product quality, and economic performance. However, the acquisition of a signal is the first and the most crucial step, and the acquired signal quality directly determines the accuracy of subsequent processes. Therefore, developing intelligent monitoring sensors for online monitoring of the cutting process has become an important issue^2^.

Various indirect sensors have been employed to acquire dynamic information^3,4^; however, all these sensor systems generally require wires for data transmission and power supply, as well as special data acquisition equipment, limiting the installation of sensor systems. Furthermore, commercial sensor systems are usually too expensive for conventional factories to use. To overcome these limitations, the design and development of integrated sensors have recently attracted the interest of several researchers.

In 1997, Santochi et al.^5^ described a new concept of cutting tools with the integration of strain gauges for sensors within the tool shank to measure the forces in turning operations. Goyal et al.^6^ fabricated a low-cost non-contact sensor system for sensing faulty bearing vibration signals. Albrecht et al.^7^ presented a method for measuring the cutting forces from the displacements of rotating spindle shafts. De Oliveira et al.^3^ and Rizal et al.^8^ designed a functional prototype of a hybrid dynamometer mounted on a newly designed force-sensing element. Liu designed and constructed a dynamometer mounted on a rotating spindle based on fibre Bragg grating^9^. Ting et al. designed and fabricated a multi-axis sensor made of polyvinylidene fluoride film^10^.

Recently, considering the advantages of piezoceramic components in terms of stiffness and sensitivity, they have been used as sensors owing to their excellent potential in miniaturization and integration of sensors for vibration control and monitoring cutting operations^11^. Qin et al.^12^ designed an integrated cutting force measurement system for measuring the axial force and torque in the milling process based on the piezoresistive micro-electromechanical system (MEMS) sensors. Chen et al.^13^ designed innovative turning tools based on the piezoelectric film. Drossel et al.^14^ presented a sensor concept based on piezoelectric film sensors mounted directly on the milling tool behind the indexable insert.

In addition to dynamometers, vibration signals are one of the most effective signals used for monitoring the machining process^15^. However, integrated sensing systems for vibration-signal acquisition have rarely been studied. Xie et al.^16^ and Zhou^17^ developed integrated wireless vibration sensing tool holders for monitoring the milling process; however, the standard tool holder should be modified to install the sensor. Chung et al.^18^ developed a wireless three-single-axis MEMS accelerometer sensing system; however, the sampling frequency was only 150 Hz, which did not meet the milling process requirements. Totis et al.^19^ and Nguyen et al.^20^ designed a smart turning tool with a sensor adhesively bonded to the tool shank for monitoring the turning process. However, it is only necessary to measure the cutting force and modify the structure of the cutting tool.

To the best of the authors’ knowledge, limited research has been performed on the development of integration wireless vibration sensors for monitoring the cutting process of lathes. Therefore, this study aimed to develop a novel three-axial wireless on-rotor sensing (ORS) accelerometer to monitor the turning process, which showed better static and dynamic characteristics in addition to being more sensitive to the vibration signals produced by the entire rotor system.

The following issues were addressed.

1. Absence of wireless vibration sensing systems for monitoring the turning processes.
2. Re-design and re-assembly problems regarding the turning tool or lathe machines.
3. Insufficient analysis of the dynamic characteristics of the system based on the vibration signals.

This study developed a novel wireless vibration model using the ORS system for monitoring the turning processes and machining conditions (such as tool wear conditions and tool insert breakage occurrence), which can simultaneously measure triaxial vibration signals with a measuring range of ± 16 g. A piezoelectric accelerometer was integrated into the designed sensor device mounted on one end of the workpiece without any tool holder or machine modifications.

## Configuration of the on-rotor sensing

### Overall configuration of the system

**Figure 1:**
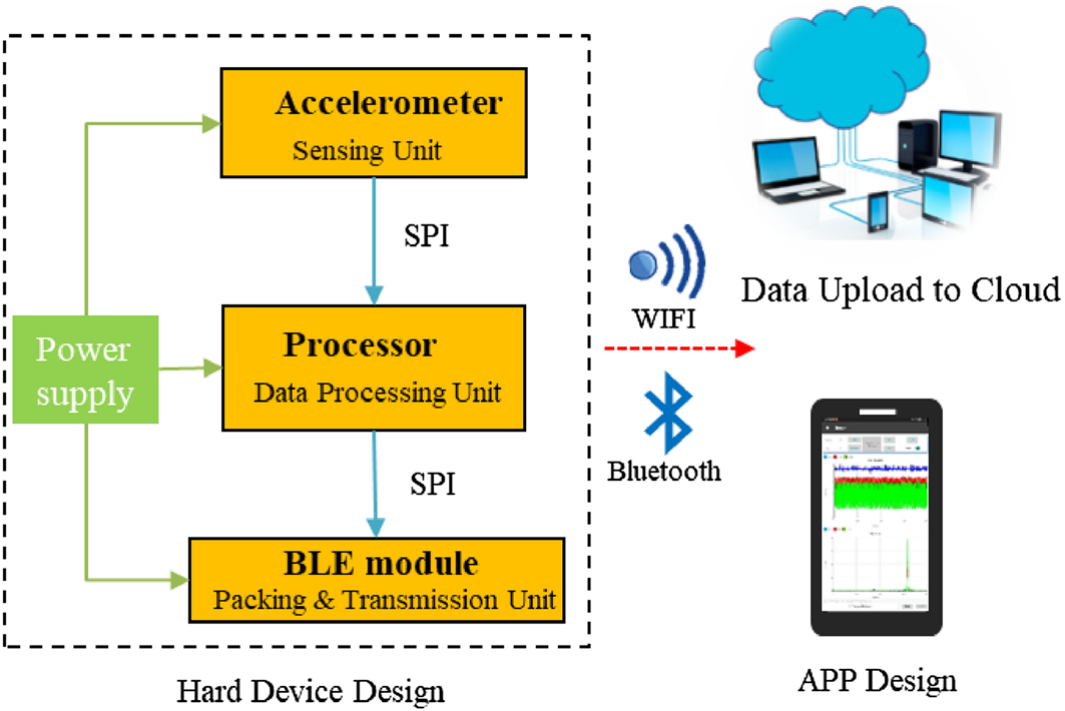
Schematic of the ORS system.

A schematic of the three-axis ORS system is shown in Fig. 1. The hardware modules included a three-axis accelerometer as the sensing unit, a processor as the data processing unit, and a BLE chip module as the packing and transmission unit. A lithium battery powered the circuits. The sensed acceleration signals were transmitted to the APP or cloud through WIFI or Bluetooth.

### Core components selection

ADXL345 is a three-axis MEMS accelerometer that offers stable packaging techniques and low noise performance of less than $290ug/\sqrt{Hz}$, along with low power consumption and cost. The sampling rate of ADXL345 is 3200 Hz, and the communication bandwidth is 1 Mbps. The other parameters of ADXL345 are listed in Assembly of the ORS.

The processing unit board and battery were integrated into a cylindrical shell as shown in Fig. 3. The sensing unit was mounted at the centre of the bottom of the cylindrical shell, which was separated from the processing unit to reduce the influence of vibrations on the processor board and battery. The cylindrical shell was manufactured using a 3D printing technology with ABS resin to ensure high transmission quality of BLE 5.0. The shell had a switch button and a charging interface port and was assembled on the connection board by fastening the bolts. A connection sleeve was used to connect the sensor to the workpiece by fastening the bolts. Consequently, the entire wireless three-axis ORS could rotate together with the target rotor system to obtain online vibration information.

**Figure 2:**
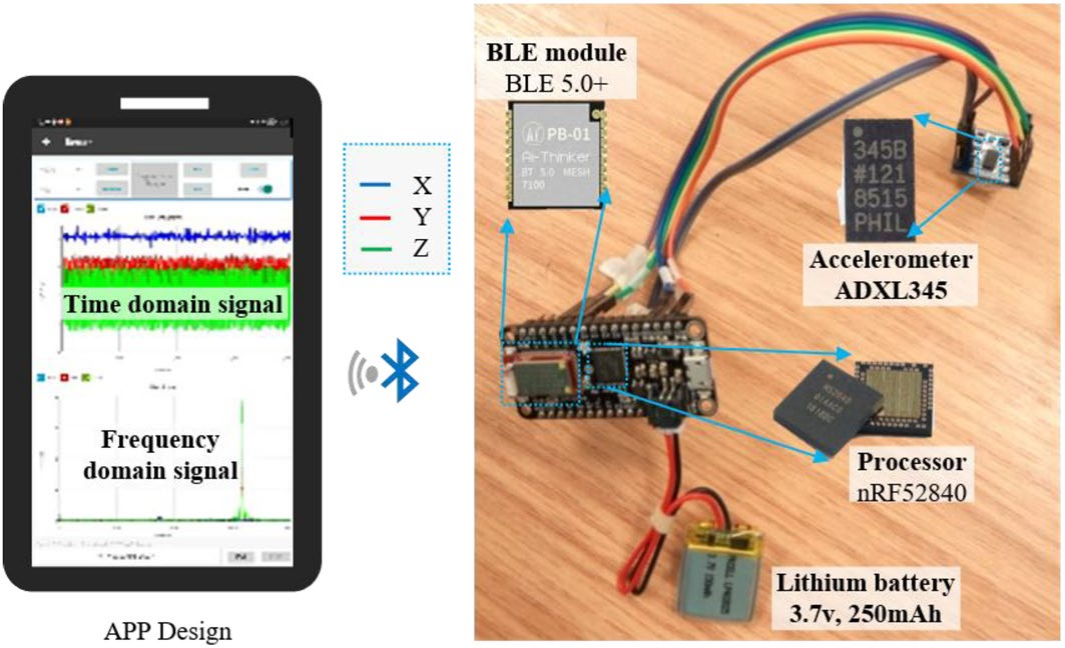
Configured components of the sensor system.

The sensing unit was connected to the processor and integrated into a separate printed circuit board (PCB), as shown in Fig. 2. The rectangle MEMS accelerometer was not designed to be at the centre of the PCB.

The digitalized signal through an analogue digital converter was transmitted to the nRF52840 processor, built on a 32-bit ARM^®^ Cortex™-M4 CPU with a full protocol concurrency such as Bluetooth LE, Bluetooth mesh, Thread, and Zigbee. In the present configuration, the digital signals were transmitted through Bluetooth LE 5.0, with a serial port baud rate of up to 1 Mbps.

The data processing unit was primarily responsible for receiving, saving, packaging, and sending the digitalized signal from the MEMS sensing unit. Meanwhile, intelligent host software was developed based on Arduino to convert hexadecimal data into decimal numbers. The vibration signals of the three-axis system were extracted, frequency analysis was performed, and the final vibration signal both in time and frequency domains in the APP was displayed.

The data processor and wireless transmission modules were integrated on a double-sided PCB to reduce the structural size and facilitate the installation. A 3.7 V lithium battery pack was used to power the entire system. The configured components of the ORS are illustrated in Fig. 2 (Table 1).

**Table 1:** Main parameters of ADXL345.

Power current (μA)	Dimensions (mm)	Measurement range (g)	Frequency range (Hz)	Resolution (g/bit)
25–130	3 × 5 × 1	± 16	0–3200	16/4096

### Assembly of the ORS

**Figure 3:**
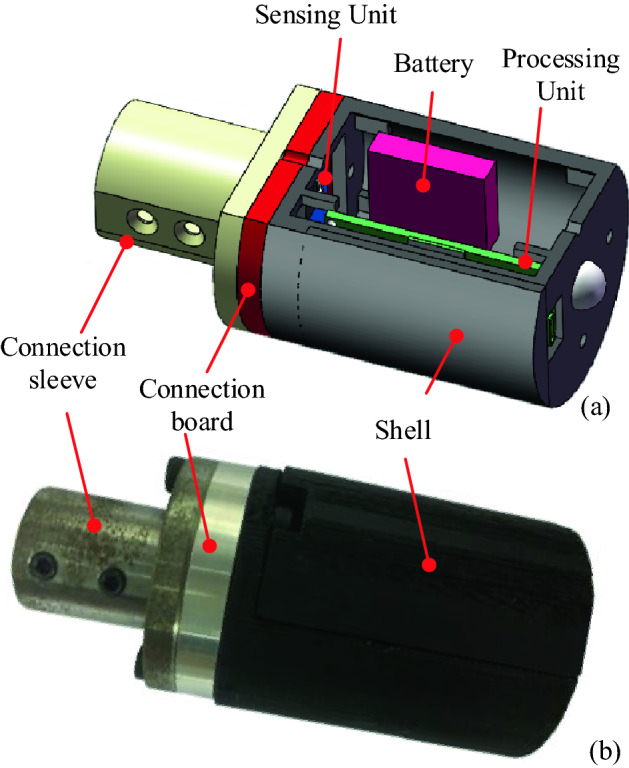
Three-dimensional illustration of the structural model: (**a**) assembly structure and (**b**) physical appearance.

The processing unit board and battery were integrated into a cylindrical shell as shown in Fig. 3. The sensing unit was mounted at the centre of the bottom of the cylindrical shell, which was separated from the processing unit to reduce the influence of vibrations on the processor board and battery. The cylindrical shell was manufactured using a 3D printing technology with ABS resin to ensure high transmission quality of BLE 5.0. The shell had a switch button and a charging interface port and was assembled on the connection board by fastening the bolts. A connection sleeve was used to connect the sensor to the workpiece by fastening the bolts. Consequently, the entire wireless three-axis ORS could rotate together with the target rotor system to obtain online vibration information.

The weight of the sensing unit was only 0.6 g, and the processing unit weighed approximately 4.2 g. The total weight of the ORS along with the connection board was 87 g when all the components are assembled in the 3D-printed cylindrical shell. The weight of the ORS is relatively small compared with that of the spindle system of the lathe and was axially and symmetrically installed on one end of the workpiece through the connection sleeve. Therefore, the design and installation of the sensor had little effect on the dynamic imbalance of the spindle system.

## Performance of the ORS

### Outputs of the ORS MEMS accelerometer

**Figure 4:**
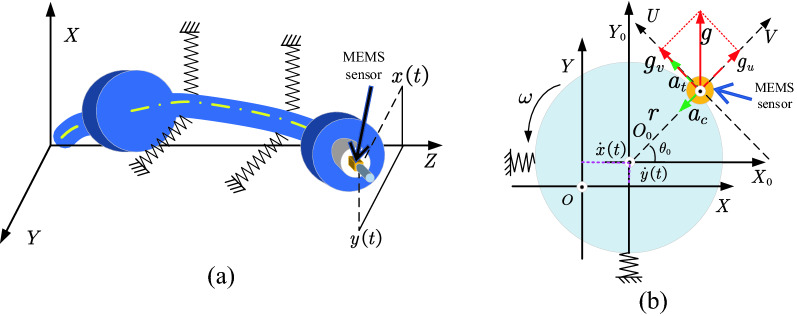
(**a**) Spindle rotor system with the ORS mounted on one end of the workpiece and (**b**) output analysis of the MEMS sensor in the rotor system.

To study the outputs of the ORS mounted on the spindle rotor system, three Cartesian coordinate systems were defined, as shown in Fig. 4. The $XYZ$ is the coordinate of the stationary frame of the rotor system, which acts as the reference for any rotating object. The ${X}_{O}{Y}_{O}{Z}_{O}$ is a rotating coordinate owing to the static bending deformation dynamic vibration of the rotating shaft under the influence of the cutting force, as shown in Fig. 4a. The centre of the rotating shaft was shifted to ${O}_{0}$, and the dynamic vibration is expressed as $\ddot{x}(t),\ddot{y}(t)$ in the fixed coordinate system $XYZ$. The $UVW$ is also a rotating coordinate, which was aligned with the positive outputs of the ORS during rotation at an angular velocity of $\omega$. The MEMS accelerometer was mounted at the end of the workpiece and assumed to have an initial phase ${\theta }_{0}$, normal to the $Z$ direction, with its *V*-direction oriented radially and *U*-direction tangentially. Furthermore, there was an offset of $r$ for the ORS because it was installed axially and symmetrically on one end of the workpiece, whereas the MEMS accelerometer was not designed to be at the centre of the PCB (Fig. 2).

As shown in Fig. 4b, both the centrifugal acceleration ${a}_{c}$ as well as radial direction and the tangential accelerometer ${a}_{t}$ could be sensed by the ORS in the *V* and *U* directions, respectively. Furthermore, it is known from the principle of the MEMS accelerometer that it generally senses an acceleration of 1.0 g in the gravitational field but in the opposite direction to the earth’s gravity. This means that there is always an acceleration of 1.0 g vertically upwards, regardless of whether the system is rotating. Therefore, the outputs of the ORS are:

1 $$\begin{aligned} & \ddot{u}(t) = - \ddot{x}(t)sin\left( {\omega t} \right) + \ddot{y}(t){\text{cos}}\left( {\omega t} \right) + g{\text{cos}}\left( {\omega t} \right) + a_{t} \\ & \ddot{v}(t) = \ddot{x}(t){\text{cos}}\left( {\omega t} \right) + \ddot{y}(t)sin\left( {\omega t} \right) + gsin\left( {\omega t} \right) + a_{c} \\ \end{aligned}$$

where ${a}_{t}$ and ${a}_{c}$ can be expressed as:

2 $$\begin{aligned} & a_{c} = r\omega ^{2} \\ & a_{t} = r\dot{\omega } \\ \end{aligned}$$

Then the matrix format for Eq. (1) is:

3 $$\left[\begin{array}{c}\ddot{u}(t)\\ \ddot{v}(t)\end{array}\right]=\left[\begin{array}{cc}-\mathrm{sin}(\omega t)& \mathrm{cos}(\omega t)\\ \mathrm{cos}(\omega t)& \mathrm{sin}(\omega t)\end{array}\right]\left[\begin{array}{c}\ddot{x}(t)\\ \ddot{y}(t)+g\end{array}\right]+\left[\begin{array}{c}r\dot{\omega }\\ r{\omega }^{2}\end{array}\right]$$

Equation (3) reveals that the measured signals consist of acceleration, which reflects the dynamic characteristics of the rotor system rotation owing to the machining operation and components of the gravitational accelerations. The latter is not the desired signal and should be eliminated to enhance proper rotor dynamic signals for identifying the cutting status^21^.

### Acceleration signal reconstruction

Assuming that the rotor rotates at a time-varying angular speed $\omega$, as $\omega ={\omega }_{0}+{\omega }^{^{\prime}}$, where ${\omega }_{0}$ is the steady angular speed and ${\omega }^{^{\prime}}$ is the fluctuating component of the speed. Then, the centripetal ${a}_{c}$ and tangential accelerations ${a}_{t}$ can be written as:

4 $$\begin{aligned} & a_{c} = \left( {\omega _{0} + \omega ^{\prime}} \right)^{2} = r\omega _{0}^{2} + 2r\omega _{0} \omega ^{\prime} + r\omega ^{{\prime 2}} \\ & a_{t} = r\frac{{d\omega ^{\prime}}}{{dt}} \\ \end{aligned}$$

Because the dynamic fluctuation of the angular speed of a rotor ${\omega }^{^{\prime}}$ is significantly small compared with that of the steady angular speed ${\omega }_{0}$, the quadratic term $r{{\omega }^{^{\prime}}}^{2}$ is negligible. Consequently, the dynamic centripetal acceleration ${a}_{c}^{^{\prime}}$ can be approximated as:

5 $${a}_{c}^{^{\prime}}\approx 2r{\omega }_{0}{\omega }^{^{\prime}}$$

Moreover, the dynamic angular speed can be considered periodic and expanded as a Fourier series as follows:

6 $${\omega }^{^{\prime}}=\sum_{n=1}^{\infty }{A}_{n}\mathrm{sin}(n{\omega }_{0}t+{\varphi }_{n})$$

where ${A}_{n}$ and ${\varphi }_{n}$ are the amplitude and phase of the *n*th harmonic, respectively.

Finally, the dynamic centripetal ${a}_{c}^{^{\prime}}$ and dynamic tangential accelerations ${a}_{t}^{^{\prime}}$ can be expressed as a combination of harmonic components as follows:

7 $$\begin{aligned} & a_{c}^{\prime } = \sum\limits_{{n = 1}}^{\infty } 2 r\omega _{0} A_{n} {\text{sin}}(n\omega _{0} t + \varphi _{n} ) \\ & a_{t}^{\prime } = \sum\limits_{{n = 1}}^{\infty } n rA_{n} {\text{cos}}(n\omega _{0} t + \varphi _{n} ) \\ \end{aligned}$$

In the ${X}_{O}{Y}_{O}{Z}_{O}$ coordinate system, the $\omega t$ can be expressed as follow:

8 $$\omega t={\theta }_{0}+{\omega }_{0}t+{\int }_{0}^{t}{\omega }^{^{\prime}}dt$$

where ${\theta }_{0}$ is the initial phase, and the third component can be neglected compared with the first two components. Subsequently, the time-varying dynamic vibration, as shown in Eq. (3) can be rearranged as

9 $$\left[ {\begin{array}{*{20}l} {\ddot{u}\left( t \right)} \\ {\ddot{v}\left( t \right)} \\ \end{array} } \right] = \left[ {\begin{array}{*{20}c} { - {\text{sin}}\left( {\theta _{0} + \omega _{0} t} \right)} & {{\text{cos}}\left( {\theta _{0} + \omega _{0} t} \right)} \\ {{\text{cos}}\left( {\theta _{0} + \omega _{0} t} \right)} & {{\text{sin}}\left( {\theta _{0} + \omega _{0} t} \right)} \\ \end{array} } \right]\left[ {\begin{array}{*{20}c} {\ddot{x}\left( t \right)} \\ {\ddot{y}\left( t \right) + g} \\ \end{array} } \right] + \left[ {\begin{array}{*{20}c} {\sum\nolimits_{{n = 1}}^{\infty } {nrA_{n} \;{\text{cos}}\;(n\omega _{0} t + \varphi _{n} )} } \\ {\sum\nolimits_{{n = 1}}^{\infty } {2r\omega _{0} A_{n} \;{\text{sin}}\;(n\omega _{0} t + \varphi _{n} )} } \\ \end{array} } \right]$$

From Eq. (9), the reconstructed dynamic acceleration $\ddot{u}\left(t\right)$ and $\ddot{v}\left(t\right)$, $\mathrm{respectively},$ projected on the *U*-axis and *V*-axis, are comprised of two components: the dynamic vibration of $\ddot{x}\left(t\right)$ and $\ddot{y}(t)$ related to the cutting operation, and the dynamic centrifugal ${a}_{c}^{^{\prime}}$ and tangential accelerations ${a}_{t}^{^{\prime}}$. When performing a Fourier transform on the vibration signals, it is necessary to eliminate the projection of gravitational acceleration components, thus permitting the reconstruction of a meaningful vibration signal of dynamic centripetal acceleration ${a}_{c}^{^{\prime}}$, dynamic tangential acceleration ${a}_{t}^{^{\prime}},$ and dynamic vibration $\ddot{x}\left(t\right)$, $\ddot{y}(t)$ owing to machining. The steps for reconstructing the acceleration signal of the rotor system are as follows.

1. Calculate and determine the position of the rotation frequency of the rotor system after the Fourier transform.
2. Subtract 1.0 g from the amplitude in the complex domains, both in the projection of *X*-direction and *Y*-direction at the rotation frequency.
3. Reconstruct the time domain signal using the inverse Fourier transform.

### Modal analysis of the spindle rotor system

**Figure 5:**
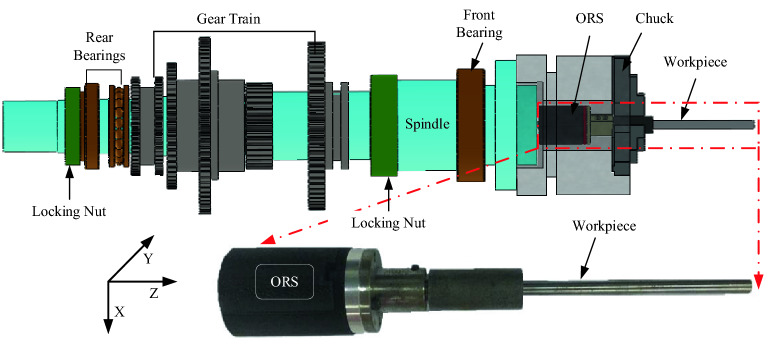
Finite element model of the spindle rotor system.

Considering that the spindle-chuck assembly has a non-negligible effect on the dynamics of the machined workpieces^22,23^, a multi–degree-of-freedom system consisting of a spindle, gears, chuck, and workpiece was established through the finite element method (FEM), as shown in Fig. 5. The front bearing group was composed of two DBB-mounted NSK 51214 and NSK 32014 bearings. The rear bearing is a double-row cylindrical roller bearing of type NSK NN3019K. The main parameters of the bearings are presented in Table 2. The stiffness was calculated using the theoretical method stated in a previous study^24^ and is presented in Table 3.

**Table 2:** Bearing parameters.

Bearing type	Contact angle $\alpha$	Inner diameter *d/mm*	Outside diameter *D/mm*	Circular diameter of roller *D*_*mp*_*/mm*	Ball diameter /Roller length *D*_*b*_*/l mm*	Number of balls (*Z*)	Cr/*N*
NSK 51214	90°	70	105	87	14	20	73.6e3
NSK 32014	15°	70	110	93	14.14	28	105e3
NSK NN3019K	0°	95	145	126	9.25*2	82	150e3

**Table 3:** Stiffness of the rolling bearing.

Bearing	Radial stiffness (N/mm)	Axial stiffness (N/mm)
NSK 51214	0	50.978 × 10^5^
NS 32014	29.449 × 10^5^	2.386 × 10^3^
NSK NN3019K	56.510 × 10^5^	0

Table 4 summarizes the modal results of the spindle rotor system. Four frequency bands were observed at approximately 46 Hz, 350–450 Hz, 750–900 Hz, and 1000–1200 Hz.

**Table 4:** Results of the modal analysis of the spindle rotor system.

Modal order	1st	2nd, 3rd	4th, 5th	6th, 7th	8th	9th
Mode of vibration	1st rigid torsional	1st transverse vibration	1st transverse vibration of workpiece	2nd transverse vibration	1st longitudinal vibration	2nd torsional vibration
Natural frequency (Hz)	46.79	365.62	755.29	881.87	913.17	1033.9

### Vibration model of the rotor system under the cutting force

As shown in Fig. 5, the spindle rotor system is axially symmetric, and its dynamic characteristics are assumed identical in the *X*- and *Y*-directions. ${F}_{x}$ and ${F}_{y}$ are the projections of the cutting force on the *X*- and *Y*-axes, respectively. The tangential force ${F}_{x}$ is the primary cutting force, accounting for more than 95% of the resultant cutting force, and the radial force ${F}_{y}$ accounts for less than 10%. Consequently, the centre of the spindle rotor shows a slight lateral swing in the *X*-direction. The dynamic orbits of the spindle rotor system then exhibit significant fluctuations compared with that of the standard circular or elliptical orbits.

The corresponding dynamic equation of the spindle rotor system excited by the cutting force is expressed as^4^

10 $$\ddot{x}\left(t\right)+2{\omega }_{n}\zeta \dot{x}\left(t\right)+{\omega }_{n}^{2}x\left(t\right)=\frac{1}{m}F(t)+\frac{1}{m}{F}_{\sigma }(t)$$

where $m,$ ${\omega }_{n},$ and $\zeta$ are the equivalent mass, natural frequency, and damping ratio, respectively, of the system. $F(t)$ denotes the instantaneous radial cutting force. ${F}_{\sigma }\left(t\right)$ is the stochastic cutting force owing to the friction or uncertainties in machining, which can excite the stochastic resonance of the machine system.

The tangential cutting force $F(t)$ can be obtained using the empirical formula^25^:

11 $$F(t)=Kch(t)w(t{)}^{q}$$

where ${K}_{c}$ is the coefficient related to the materials of the tool and workpiece and cutting parameters, $h\left(t\right)$ is the actual cutting thickness, also known as the depth of the cut, $w(t)$ is the actual cutting width, and $q$ denotes the exponent that can be determined experimentally. The parameters of $h\left(t\right)$ and $w(t)$ are related to the instantaneous fluctuation of vibrations in the *XOZ* plane and can be expressed as^4^:

12 $$\begin{aligned} & w(t) = z_{0} - z(t) - z\left( {t - \frac{{2\pi }}{\omega }} \right) \\ & h(t) = x_{0} - x(t) - x\left( {t - \frac{{2\pi }}{\omega }} \right) \\ \end{aligned}$$

where terms ${x}_{0}$ and ${z}_{0}$ are the original cutting depth and cutting width, respectively. $x(t)-x(t-\frac{2\pi }{\omega })$ denotes the fluctuation of the dynamic chip thickness produced by radial vibrations, and $z(t)-z\left(t-\frac{2\pi }{\omega }\right)$ is the fluctuation of the instantaneous chip width produced by axial vibrations.

From Eqs. (9), (10), and (12), it is known that transient variations in cutting parameters such as depth of cuts, cutting force, and axis orbits can be reflected through the fluctuation of acceleration that the ORS senses.

## Experimental evaluation and discussion

### Experimental setup

**Figure 6:**
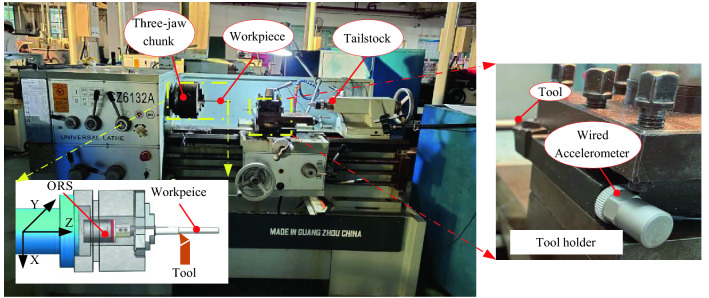
Experimental setup.

To verify the performance of the vibration signal acquired from the ORS in a practical machining process, turning experiments were conducted on a universal horizontal lathe (CZ6132A). Both the ORS and traditional wired acceleration sensor were used for data acquisition, as shown in Fig. 6. The ORS was mounted on one end of the workpiece, which was placed through the hole of a three-jaw chuck, and its vibration signal could be transmitted to the APP on a phone or other intelligent terminal. However, the wired accelerometer (122A200, with a sensitivity of 10.2 m^2^/s produced by the YMC Piezotronics Inc) was fixed on the tool holder at a sampling rate of 100 kHz. The other cutting parameters are presented in Table 5.

**Table 5:** Main parameters of the experiments.

Parameters	Specific parameters	Specific values
Cutting parameters	Rotational speed	1080 r/min
	Depth of cut	0.25 mm × 0.5 mm × 0.75 mm
Workpiece parameters	Material	Q235
	Initial diameter	22 mm
	Total length	215 mm
	Cutting length	135 mm

As shown in Fig. 6, the vibration signals projected in the *X, Y,* and *Z* directions can be directly sensed by the developed ORS. However, the single-axis wired accelerometer only showed the vibration in the radial cutting force direction, and it could not capture the dynamic characteristic of the spindle rotor. The workpiece was machined from 21.9 to 10 mm. The turning experiment was performed continuously, with experimental data recorded for each cut. It was then moved to the next layer cut until the workpiece was cut to approximately 10 mm.

### Comparison of the signals from ORS and wired accelerometer

**Figure 7:**
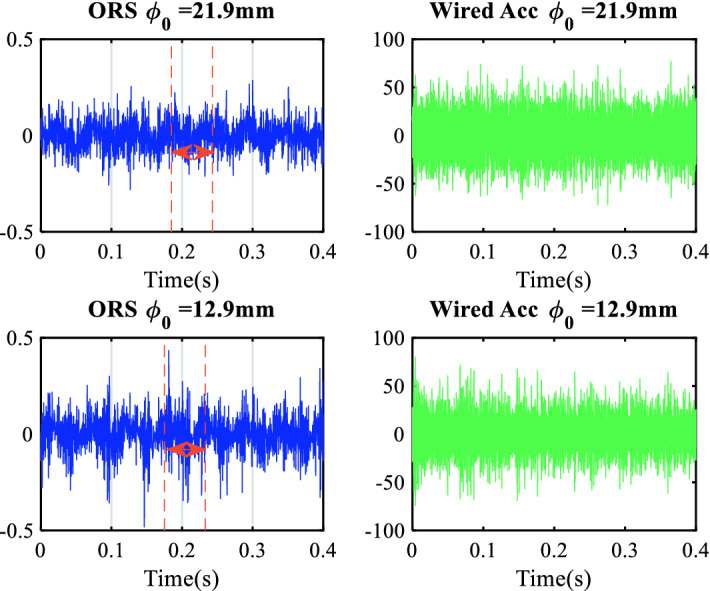
Comparison of vibration signals in the time domain.

**Figure 8:**
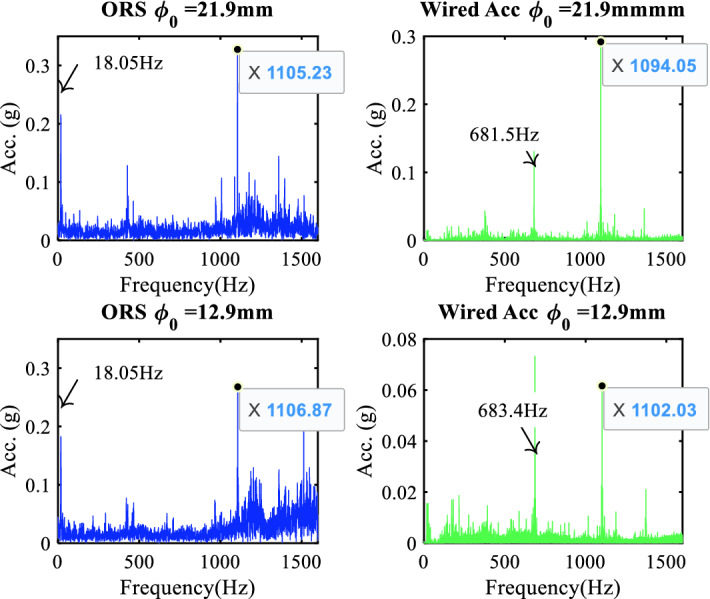
Comparison of vibration signals in the frequency domain.

Figure 7 shows the comparison of the vibration signals collected from the ORS and wired accelerometer in the time domain of 0.4 s when the cutting depth was 0.5 mm. Figure 8 shows the same signal in the frequency domain after reconstruction, following the steps described in “Acceleration signal reconstruction”.

The spectrum shown in Fig. 8 from the ORS was primarily modulated by a spindle rotational frequency of 18 Hz, which had a significantly larger amplitude than that generated by the machining vibration. Conversely, this signal coupling collected from the wired accelerometer was drowned in the noise. There were three evident frequency bands with significantly larger amplitudes, which showed the resonant phenomenon excited by the random signal. The resonant frequency bands ranged from 350 to 480 Hz, 600–700 Hz, and 1000–1200 Hz, consistent with the FEM results. These vibrations of approximately 680 Hz were detected only by the wired accelerometer. This spectrum represents the transverse vibration of the workpiece at the free end (presented in Table 4 and described in “Modal analysis of the spindle rotor system”) and could be captured only by the wired accelerometer installed on the tool holder beside the free end of the workpiece; whereas, the ORS fixed on the clamping end and put in the chuck hold.

### Orbit of the spindle rotors

**Figure 9:**
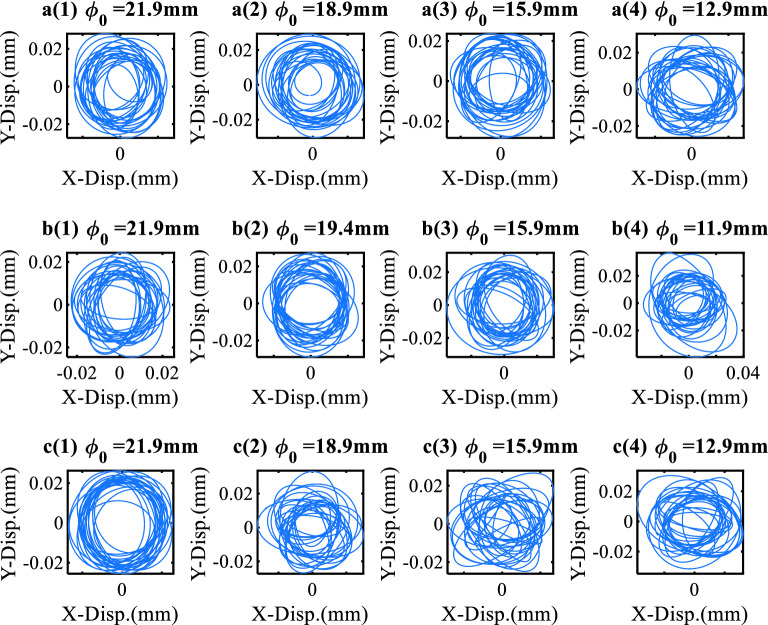
Orbit of the spindle rotors.

The axis orbit is the motion trajectory of the rotor axis and is usually composed of displacement signals in two directions at an angle of 90° relative to each other. As described above, the developed ORS could sense the acceleration in three directions and rotate with the rotor system such that it could draw the spindle rotor trajectory easily compared with the wired acceleration sensor. Furthermore, an axis orbit can directly reflect the operating conditions of a rotor system and is widely used to monitor rotor conditions and faulty diagnoses^26^. The orbit of the spindle rotor system was calculated based on this phenomenon, as shown in Fig. 9.

After eliminating the gravity of 1.0 g and reconstructing the acceleration data in the time domain, as mentioned above, the Chebyshev filter was used to filter the rotation frequency after the Fourier transform. The acceleration data in *X*- and *Y*-directions were then integrated into the frequency domain. As shown in Fig. 9, the corresponding trajectory shows a non-repetitive circuit similar to the quasi-periodic motion.

Figure 9a(1)–(4) shows the orbit of the cutting depth of 0.5 mm, b(1)–(4) shows the orbit of the cutting depth of 1.0 mm, and c(1)–(4) shows the orbit of the cutting depth of 1.5 mm. The diameter decreases as shown in figures from (1) to (4), which is simulated during the cutting process. The fluctuation of the axis trajectory increases as the diameter of the workpiece decreases. Additionally, it shows that the greater the depth of the cut, the more elliptical and chaotic the orbit is, indicating that the cutting parameters could be recognized through the orbits.

### Recognition of the cutting parameters

To monitor the cutting process and recognize the different depths of the cut, two bandpass filters were adopted based on the spectrum characteristics described in “Comparison of the signals from ORS and wired accelerometer”. For the vibration signal collected by the ORS, the first resonant frequency band was set from 200 to 600 Hz, which was consistent with the impact test results, and the second resonant frequency band was set from 800 to 1200 Hz. For the data collected by the wired accelerometer, two bandpass filters were set from 400 to 800 Hz and 7000–8500 Hz separately, which is significantly larger than that from the ORS.

**Figure 10:**
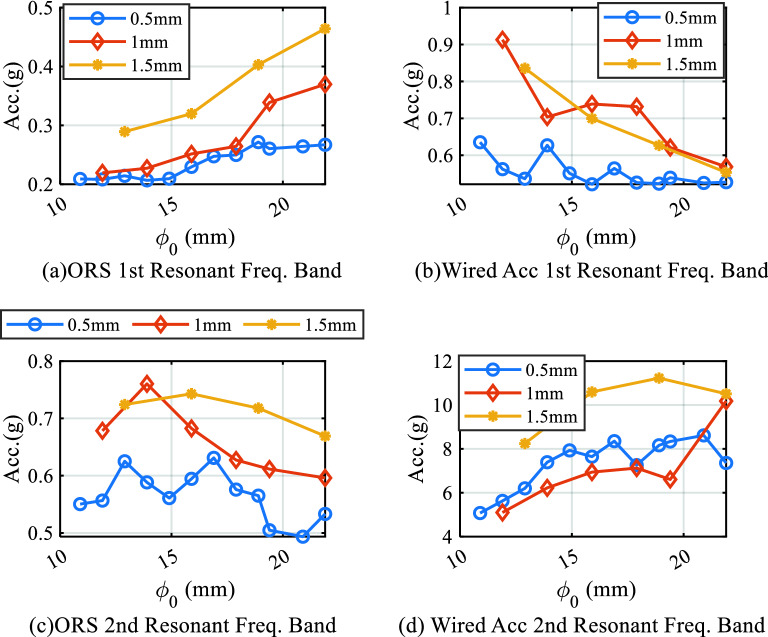
RMS under different cutting depths.

After applying the bandpass frequency filter, the Root Mean Square (RMS) was calculated at each cut. Figure 10 shows the comparison results, which suggest that different cutting depths can be recognized by the vibration signal from the ORS, both filtered by the first and second resonant frequency bandpass filters. In contrast, the vibration data from the wired accelerometer does not distinguish between different cutting depths, which showed that the proposed ORS is more effective despite their varying trends.

The main reason for obtaining different trends of data was the different mounting positions of the sensors. The wired accelerometer was installed on the tool holder and, therefore, the vibration signal was highly affected by the whole tool holder system; however, the ORS was mounted on the workpiece and rotated synchronously with the spindle; thus, it was affected by the spindle system. In conclusion, owing to the different external vibration excitations and dynamic characteristics of the two sensors, the results showed different trends.

These results proved that the developed vibration-measuring three-axis ORS system can detect changes in the vibration signal under different cutting depths more effectively and sensitively than a wired accelerometer.

## Conclusion

In this study, a novel three-axis wireless on-rotor vibration sensing system for monitoring the turning process was developed. Then, based on the outputs of the ORS MEMS accelerometer, we reconstructed the vibration signal using the inverse Fourier transform after subtracting 1.0 g from the amplitude in the complex domains. Furthermore, generally, we conducted the turning experiments on a universal horizontal lathe to verify the performance of the vibration signal acquired from the ORS and compare the results with that from the commercial wired accelerometer. Some conclusions are summarised as following.

1. A novel three-axis wireless is developed and constructed in this study for turning processing monitoring, with the sampling rate of 3200 Hz which meets the commonly requirement of cutting machine.
2. The sensing system can be mounted on one end of the workpiece such that it is more sensitive to the cutting parameters and the entire rotor system without any modification to the lathe system or interference in the cutting procedure.
3. The machining experiment showed that the developed ORS could measure the vibration signal of the rotor system more effectively and sensitively than the commercial wired accelerometer.
4. In these turning experiments, only the acceleration data in *X*- and *Y*-directions were analysed, but the vibration signal in the three directions of the three-axis ORS could be applied in more complicated machining system such as drilling procedure. In the future, studies focussing on developing and integrating various sensors to manufacture machine systems for further cutting-condition recognition and machining process monitoring.

In addition, there are some limitations of the current ORS such as a large communication bandwidth and high-power consumption. We will explore energy harvesting system and edge computing in the further to address these problems. In this way, data processing and feature analysis can be performed on the processing unite, and only the results are required to be transmitted to the APP, which reduces data transmission, bandwidth, and energy consumption and such realizing online monitoring. Furthermore, to solve the problem of battery charge, we have been studying some research on an effective prototype energy harvester method, which would enable battery charging. The wireless ORS system is expected to be self-powered in the future.

## Data Availability

The datasets used and analysed during the study are available from the corresponding author on reasonable request.
